# Varicocele Treated by Soluble Antiseptic Ligatures

**Published:** 1869-08

**Authors:** E. Andrews

**Affiliations:** Prof. of Principles and Practice of Surgery, Chicago Medical College; 81 Monroe Street


### CHICAGO MEDICAL EXAMINER.

N. S. DAVIS, M.D., Editor.

VOL. X. AUGUST, 1869. NO. 8.

### Original Contributions.

ARTICLE XXVIII.

### VARICOCELE TREATED BY SOLUBLE ANTISEPTIC LIGATURES.

By E. ANDREWS, M.D., Prof. of Principles and Practice of Surgery, Chicago Medical College.

Prof. Lister, of Glasgow, Scotland, gives an account in a late London *Lancet* of an experiment upon a calf, in which he tied the carotid artery in two places with animal ligatures, soaked in carbolic acid solution. One of the ligatures was a piece of fine catgut, and the other a string made by twisting up strips of calf's peritonæum. The ligatures were cut off close and left in the wound. The whole wound was then well washed with solution of carbolic acid, closed with sutures, and sealed up, air-tight, with a carbolic acid dressing, covered with tin foil, and healed by first intention. The Editor of the *Lancet*, in commenting on this experiment, makes the remarkable, but erroneous statement, that the dead animal ligatures employed acquired bloodvessels, etc., and actually became living organized tissues. This, however, is a laughable misconception. Professor Lister states simply this:—At the end of a month the calf was killed, and the parts carefully dissected. The incision had healed without suppuration, nor was there a particle of pus at the places of the ligatures. The artery was found perfectly closed, though the proximal ligature was close to a large branch. At first glance, it *seemed* as though the ligatures had become organized, because their places were occupied by bands of living, fibrous tissue, running round the vessel, and giving it great strength and security, notwithstanding the proximity of the large arterial branch. Close examination, however, showed that the bands consisted of new tissue, simply occupying the places of the ligatures which had become gradually absorbed. On the posterior side of the artery, a small piece of each ligature remained, whose absorption had not yet been completed.

On reflection, it seemed to me that this principle should be available for the treatment of varicose veins. The operation upon such veins by ligature has been justly dreaded on account of the occasional deaths from the septic form of phlebitis, and consequent pyæmia. The ordinary ligature, extending from the vessel to the external air, makes a deep, narrow fistula, in which the pus decomposes and gives origin to the usual poison, which soon transmits the unhealthy septic influence to the veins and to the whole system, unless the patient is peculiarly plastic in his constitution. It occurred to me that if antiseptic ligatures could be put on, of a nature that admitted of their being ultimately absorbed, without suppuration, all danger of purulent infection would be avoided; for it is to be remarked that a simple plastic inflammation of the veins has no tendency to infect the system, and is not at all dangerous.

To test the question, I devised an operation where results seem to me to be eminently satisfactory. I took a case of varicocele, and proceeded as follows:—

I prepared a number of ligatures by stripping up a piece of fresh tendon from the foreleg of an ox. These I threaded into large surgical needles, and dropped them into a watery solution of carbolic acid, containing about 20 grains to the ounce. Separating the veins from the vas deferens in the usual way, I passed a needle and ligature from before backward, between the two, bringing the needle out behind the scrotum; then, pressing the veins inward, I reinserted the needle into the same hole, and carrying it this time on the outer side of the veins, I brought it out of the original front opening, as in a well-known method of ligating the veins. I had thus an animal carbolized ligature surrounding the veins, with both ends returning from the same orifice. I now tied a knot, trying, as I drew it, to make it sink in through the opening. Here occurred a difficulty. Tendon is not as strong as silk, and hence the ligature has to be proportionably larger, from which cause the ties were so bulky that there was difficulty in causing them to retire into the opening made by the needle. I was obliged to enlarge the orifice with a bistoury, and then succeeded in making each tie sink through the opening, so that I got a square knot upon the vessels, and cut off the tendon close to them. I now found that I could make the knot disappear from sight entirely and close the skin over it. A second ligature was applied in the same way. I washed all the orifices with a 20-grain solution of carbolic acid, and sealed them up by applying collodion, in which was dissolved 10 per cent. of carbolic acid. The ordinary inflammation occurred, but of a plastic character, and without suppuration. After a week, the collodion gradually loosened its hold, and the orifices were found closed by first intention. The veins were evidently obliterated, but swollen and tender, as usual. The knots could be felt in the interior, surrounded by plastic lymph; but no abscesses occurred. The soreness subsided in about the usual time, and the ligatures remained without producing any trouble, and are doubtless now undergoing the process of absorption. It should be stated that the strength of the carbolized collodion irritated the skin at one point and raised a semi-suppurative blister; but the puncture was nevertheless found closed by first intention, and not suppurating at all. I deem this experiment upon the tying of veins to be of great importance; and if future trials shall yield similar results it will enable us to operate upon them with perfect impunity.

It is evident that the ordinary surgical needle does not cut an orifice wide enough for tendinous ligatures. I have ordered some made with blades three times the ordinary width. Doubtless, however, the same result could be obtained by making the anterior orifice in the skin by a narrow scalpel, and then passing the ordinary needle through the wound. It is important to remove the hairs from the scrotum before commencing, otherwise they will be entangled in the knots. Finally, let it be borne in mind that pyæmia rarely comes on in surgical cases, unless the patient is predisposed to it by lying in close, insufficiently ventilated apartments. Surgical patients require for their safety a constant current of fresh air flowing over them; and the fear which we have in pulmonary cases of exposing them to a draft is out of place in surgery. The idea that wounds "catch cold" is a vulgar prejudice, which our late army experience has effectually exploded.

*81 Monroe Street.*

